# Modeling the Heterogeneity of Dengue Transmission in a City

**DOI:** 10.3390/ijerph15061128

**Published:** 2018-05-31

**Authors:** Lingcai Kong, Jinfeng Wang, Zhongjie Li, Shengjie Lai, Qiyong Liu, Haixia Wu, Weizhong Yang

**Affiliations:** 1 Department of Mathematics and Physics, North China Electric Power University; Baoding 071003, China; konglc@lreis.ac.cn; 2 State Key Laboratory of Resources and Environmental Information System, Institute of Geographic Sciences and Natural Resources Research, Chinese Academy of Sciences; Beijing 100864, China; 3 Key Laboratory of Surveillance and Early-Warning on Infectious Disease, Chinese Center for Disease Control and Prevention, Beijing 102206, China; lizhongjiecdc@163.com (Z.L.); yangwz@chinacdc.cn (W.Y.); 4 WorldPop, Department of Geography and Environment, University of Southampton, Southampton SO17 IBJ, UK; laishengjie@foxmail.com; 5 Key Laboratory of Public Health Safety, Ministry of Education, School of Public Health, Fudan University, Shanghai 200433, China; 6 Flowminder Foundation, Roslagsgatan 17, SE-11355 Stockholm, Sweden; 7 State Key Laboratory for Infectious Disease Prevention and Control, Collaborative Innovation Center for Diagnosis and Treatment of Infectious Diseases, National Institute for Communicable Disease Control and Prevention, Chinese Center for Disease Control and Prevention, Beijing 102206, China; liuqiyong@icdc.cn (Q.L.); wuhaixia@icdc.cn (H.W.); 8 WHO Collaborating Center for Vector Surveillance and Management, Beijing 102206, China

**Keywords:** dengue fever, heterogeneity, transmission terms, negative binomial distribution

## Abstract

Dengue fever is one of the most important vector-borne diseases in the world, and modeling its transmission dynamics allows for determining the key influence factors and helps to perform interventions. The heterogeneity of mosquito bites of humans during the spread of dengue virus is an important factor that should be considered when modeling the dynamics. However, traditional models generally assumed homogeneous mixing between humans and vectors, which is inconsistent with reality. In this study, we proposed a compartmental model with negative binomial distribution transmission terms to model this heterogeneity at the population level. By including the aquatic stage of mosquitoes and incorporating the impacts of the environment and climate factors, an extended model was used to simulate the 2014 dengue outbreak in Guangzhou, China, and to simulate the spread of dengue in different scenarios. The results showed that a high level of heterogeneity can result in a small peak size in an outbreak. As the level of heterogeneity decreases, the transmission dynamics approximate the dynamics predicted by the corresponding homogeneous mixing model. The simulation results from different scenarios showed that performing interventions early and decreasing the carrying capacity for mosquitoes are necessary for preventing and controlling dengue epidemics. This study contributes to a better understanding of the impact of heterogeneity during the spread of dengue virus.

## 1. Introduction

Dengue fever (DF) is caused by four serotypes of dengue virus (DENV-1 to DENV-4), which can be transmitted by female mosquitoes. The major vector is *Aedes aegypti*, whereas *Aedes albopictus* acts as a secondary vector. Infection by one serotype of DENV induces lifelong immunity to the infecting serotype but only temporary partial immunity to the other three serotypes [1].

Dengue has become the most important arthropod-borne viral disease affecting humans [1], and its incidence has substantially increased worldwide in recent decades. It was estimated that there are 390 million dengue infections per year, of which 96 million manifest clinically (any level of disease severity) [2,3]. In another study, Brady et al. estimated that a total of 3.97 billion people in 128 countries are at risk of infection with DENV [3,4]. The drivers for the rapid dengue expansion include urbanization, globalization (such as travel and trade), lack of effective mosquito control, and climate change, among others [5,6].

Mathematical models have been used to model the transmission dynamics of dengue virus and to evaluate the effectiveness and/or cost effectiveness of interventions [7,8,9]. Most of these models were compartmental-type models, which generally assumed a homogeneous mixing for the mosquito bites of humans, i.e., each mosquito can bite any host with equal probability. However, this assumption was in conflict with real epidemics, which are highly heterogeneous with multiple levels: spatial heterogeneity and individual heterogeneity [10]. The spatial heterogeneity was largely governed by environmental conditions and ecological variations, causing poorly mixed mosquito-host encounters [9]. Specifically, the heterogeneous exposure of hosts to mosquito bites was due to factors such as proximity to the aquatic habitats of immature mosquitoes, the type of house [11], insecticide use [10], human movements [12,13], and so forth. The individual heterogeneity has been described in terms of heterogeneous biting, i.e., a given mosquito bites some hosts more than others [14], which was associated with human sex, age, size, pregnancy, defensive behavior, blood type, and variability in human odors, among others [10,11]. The impacts of heterogeneity on transmission dynamics have previously been addressed using meta-population models [15,16] and agent-based models [17,18,19]. However, this topic still deserves more attention [9].

The negative binomial distribution (NBD) has been widely used to represent count data, particularly for data exhibiting overdispersion. In the biological literature, NBD was used in different transmission dynamics models to explore the influence of transmission heterogeneity, including host–parasitoid models [20,21], insect–pathogen models [22], and so forth. Recently, the application of NBD was extended to epidemiological models. In a famous study, the authors integrated branching process theory and contact tracing data by assuming that the number of secondary cases resulting from each infection was described by an ’offspring distribution’ to analyze the influence of individual variations in infectiousness on disease emergence [23]. The results showed that the NBD ($R_{0}$, *k*), where the parameter *k* captures the skew in the transmission distribution, was favorable for a number of disease datasets. The values of *k* estimated from the data ranged from 0.01 to approximately 0.1, highlighting the large variance in individual infectiousness. Kucharski et al. used the same method as in [23] to examine the level of heterogeneity in the transmission of Middle East respiratory syndrome coronavirus in South Korea in 2015, and they found substantial potential for superspreading [24]. The NBD was also used to explore the spread of DF. Padmanabha et al. used a multinomial negative binomial model to describe the number of secondary infections to evaluate the overall effects of mosquito production and household human density on dengue transmission [25]. The simulation results showed that the intervention of super-productive containers was substantially more effective in reducing the risk of dengue at higher human densities. In addition, the NBD was also used in dynamics modeling for infectious diseases. Barlow used the NBD transmission function $k\ln(1+\frac{\beta I}{k})$ to model a possum-tuberculosis (TB) system, where *k* denotes the level of heterogeneity and β is the transmission rate [26]. Kong et al. developed an SEIR model with an NBD transmission function, $k\ln(1+\frac{\beta I}{kN})S$, to model the heterogeneity of contact rate for direct infectious disease [27]. The influence of different transmission functions was studied by Hoch et al. [28].

In this paper, we modeled the heterogeneity of mosquito bites of humans using a compartmental model with NBD transmission functions. The impact of heterogeneity on the transmission dynamics of DF was explored through numerical simulations. Then, we extended the model by including the aquatic phase of mosquitoes and integrating the environment and climate impacts on them. The extended model was used to reproduce the 2014 DF outbreak in Guangzhou, China. Finally, we analyzed the impacts of different interventions on the transmission of DF through simulations in different scenarios.

## 2. Materials and Methods

### 2.1. Data

**Figure 1:**
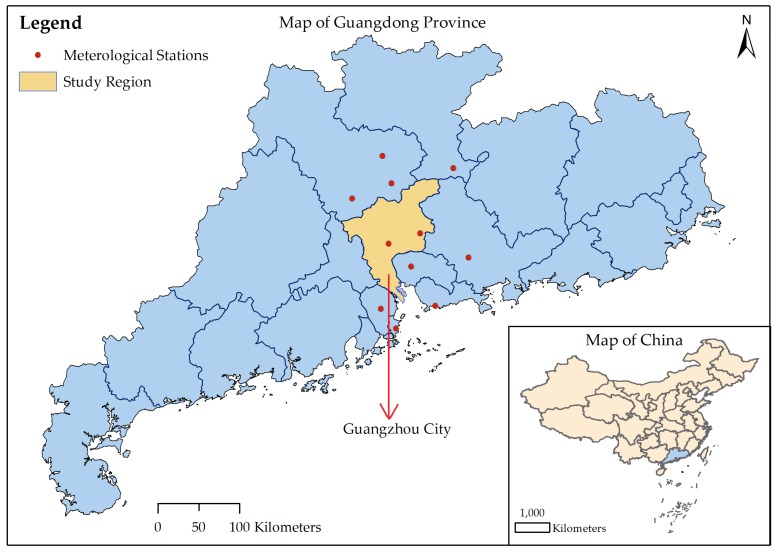
Geographic locations of Guangdong Province, the city of Guangzhou and the meteorological stations used to interpolate the climate data for Guangzhou.

**Figure 2:**
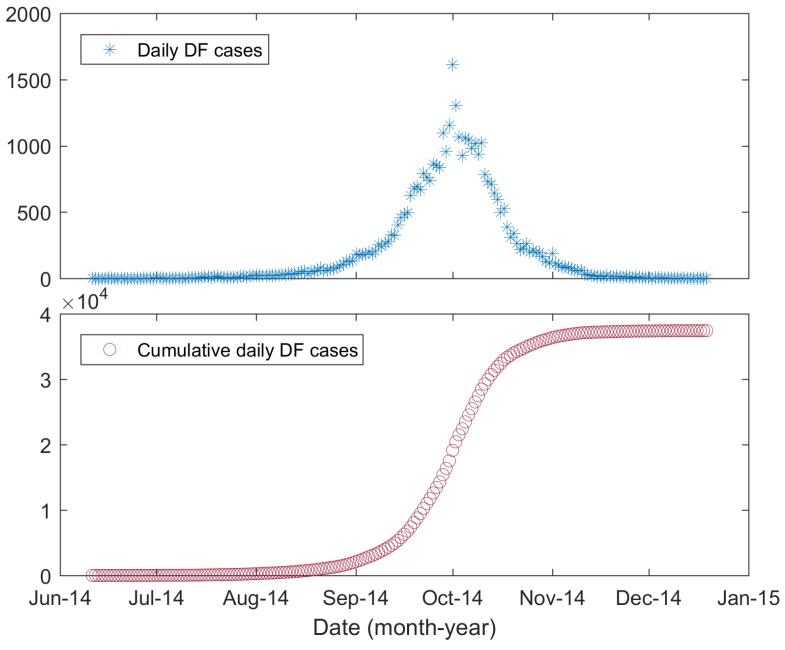
Daily reported and cumulative DF cases in Guangzhou, 2014.

In 2014, an unanticipated severe DF outbreak occurred in Guangdong Province, with more than 45,000 cases of infection [29,30]. As the provincial capital of Guangdong Province, Guangzhou was the most affected city, with 37,420 reported local DF cases. In this paper, we chose Guangzhou as our study region, and its geographic location is shown in Figure 1. DF outbreaks in China were previously thought to be imported [29,31], although recent studies suggested that DF may be endemic to China [32,33]. Here, we only considered the local DF cases and considered them to be triggered by imported cases [31]. Figure 2 shows the daily new DF cases and cumulative cases. The data were collected based on the onset date in the case data obtained from the Chinese Center for Disease Control and Prevention (CDC, http://www.chinacdc.cn/). The first reported local DF case occurred on 11 June 2014, followed by sporadic cases until the middle of July; then, the number of cases gradually increased. A rapid increase in DF cases occurred in September, and the number of cases peaked in early October. After 11 October, the number of DF cases gradually decreased. The last case of DF was reported on 19 December 2014.

**Figure 3:**
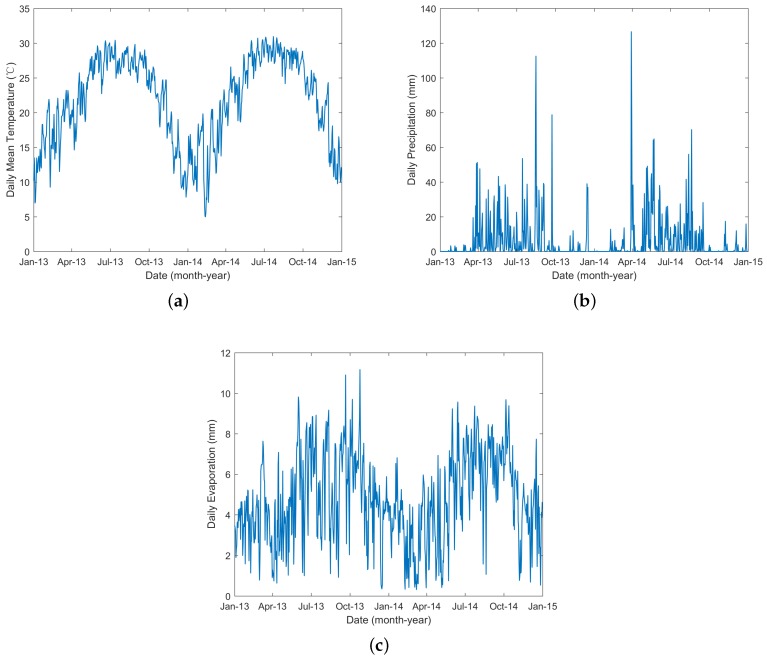
Daily temperature, precipitation and evaporation data from 2013 to 2014 for Guangzhou.

The main mosquito that transmitted dengue in Guangzhou, China, was *Aedes albopictus* [34,35], and the population of this species was influenced by climate. The temperature, precipitation and evaporation data from 2013 to 2014 were obtained from the China Meteorological Data Sharing Service System (http://data.cma.cn/). These data were monitored by meteorological stations located in Guangzhou and within 50 kilometers of the borders of Guangzhou (Figure 1). Then, the inverse distance weighted interpolation method [36] was employed to obtain the corresponding data for Guangzhou. The interpolation results (shown in Figure 3) were used as inputs to the following models.

### 2.2. The NBD SEIR-SEI Model

**Figure 4:**
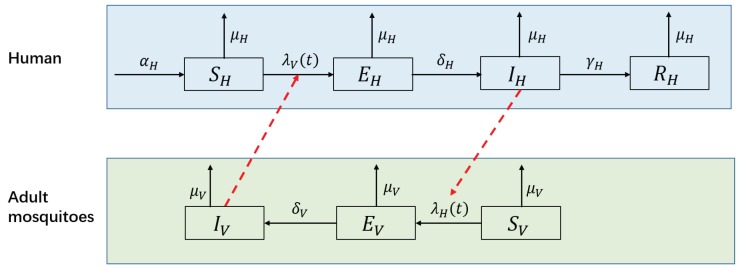
Flow diagram of the SEIR-SEI model of human-vector interactions.

A number of mathematical models have been developed to understand the dynamics of dengue infection and to evaluate the effectiveness and/or cost effectiveness of control strategies [7,8]. Among these models, the SEIR(human)–SEI(vector) compartmental model has been widely used to model the transmission dynamics of a single serotype of DENV [7,8,37,38,39,40]. This type of model divides humans into susceptible, exposed, infectious and removed groups and divides mosquitoes into susceptible, exposed and infectious groups. DENV is passed to susceptible humans ($S_{H}$) through the bites of infectious female mosquitoes ($I_{V}$); the infected humans then enter the exposed compartment $E_{H}$. After an incubation period of 4 to 10 days [41], the patients enter compartment $I_{H}$ and can transmit the virus (for 4–5 days, maximum of 12 days [3]; in another study, 1–7 days, mean of 4.5 days [42]) via mosquito bites. Finally, the hosts become immune to that serotype and enter compartment $R_{H}$. Susceptible mosquitoes ($S_{V}$) are infected by biting infectious humans ($I_{H}$), and then they enter the exposed compartment $E_{V}$. After the extrinsic incubation period (EIP) of 8 to 12 days [41], infected mosquitoes are capable of transmitting the virus for the remainder of their life ($I_{V}$). Figure 4 presents the structure of this model, and Table 1 shows the biological meanings, ranges and values of the parameters used in the following simulation.

Most of these models assumed that the mosquito-host encounters were well mixed, i.e., any given host had an equal probability of being bitten by any given mosquito [7,8]. This assumption resulted in a well-mixed transmission function between infectious mosquitoes and susceptible human hosts, with the form:

(1) $$\frac{ap_{V}I_{V}}{N_{H}}S_{H}$$

and that between infectious human hosts and susceptible mosquitoes:

(2) $$\frac{ap_{H}I_{H}}{N_{H}}S_{V}.$$

However, this assumption is particularly unrealistic at large spatial scales (for example, a whole city): a given mosquito may only have the opportunity to bite a limited subset of hosts in the population, i.e., the numbers of bites of different hosts by any given mosquito differ. We assumed that the number of effective bites (bites causing the transmission of DENV) of the *i*th susceptible human from infectious mosquitoes, $X_{i}$, followed a Poisson distribution with parameter $\theta_{i}$. Unique values for all $\theta_{i}$ results in the homogeneous mixing model. Here, we assumed that the values of all $\theta_{i}$ were different from person to person and that it followed a Gamma distribution with shape parameter $k_{1}$ and a scale parameter. After some derivations [27], we obtained the NBD transmission function from infectious mosquitoes to susceptible humans:

(3) $$k_{1}\ln\left(1+\frac{ap_{V}I_{V}}{k_{1}N_{H}}\right)S_{H},$$

where $k_{1}$ characterized the level of heterogeneity of bites of susceptible humans by infectious mosquitoes.

Similarly, assuming that the number of effective bites of infectious hosts from the *j*th susceptible mosquito, $Y_{j}$, also followed a Poisson distribution with parameter $\delta_{j}$, which had a Gamma distribution with shape parameter $k_{2}$, we obtained the NBD transmission function from infectious humans to susceptible mosquitoes:

(4) $$k_{2}\ln\left(1+\frac{ap_{H}I_{H}}{k_{2}N_{H}}\right)S_{V},$$

where $k_{2}$ characterized the level of heterogeneity of bites of infectious humans by susceptible mosquitoes.

Because $k_{1}$ and $k_{2}$ both characterized the level of heterogeneity of mosquito bites of humans, we assumed that $k_{1}=k_{2}=k$ for simplicity. With the derived NBD transmission functions (Equations (3) and (4)), we constructed an NBD SEIR-SEI compartmental model to describe the transmission process of DENV. This model can be represented by a set of ordinary differential equations (Equation (5)), in which the human and vector populations were assumed to be constants:

(5) $$\left\{\begin{array}{cc} \frac{dS_{H}}{dt} & =\mu_{H}N_{H}-k\ln\left(1+\frac{ap_{V}I_{V}}{kN_{H}}\right)S_{H}-\mu_{H}S_{H},\\ \frac{dE_{H}}{dt} & =k\ln\left(1+\frac{ap_{V}I_{V}}{kN_{H}}\right)S_{H}-\left(\delta_{H}+\mu_{H}\right)E_{H},\\ \frac{dI_{H}}{dt} & =\delta_{H}E_{H}-\left(\gamma_{H}+\mu_{H}\right)I_{H},\\ \frac{dR_{H}}{dt} & =\gamma_{H}I_{H}-\mu_{H}R_{H},\\ \frac{dS_{V}}{dt} & =\mu_{V}N_{V}-k\ln\left(1+\frac{ap_{H}I_{H}}{kN_{H}}\right)S_{V}-\mu_{V}S_{V},\\ \frac{dE_{V}}{dt} & =k\ln\left(1+\frac{ap_{H}I_{H}}{kN_{H}}\right)S_{V}-\left(\delta_{V}+\mu_{V}\right)E_{V},\\ \frac{dI_{V}}{dt} & =\delta_{V}E_{V}-\mu_{V}I_{V}. \end{array}\right.$$

Because

$$\lim_{k\to \infty }k\ln\left(1+\frac{ap_{V}I_{V}}{kN_{H}}\right)S_{H}=\frac{ap_{V}I_{V}}{N_{H}}S_{H},$$

$$\lim_{k\to \infty }k\ln\left(1+\frac{ap_{H}I_{H}}{kN_{H}}\right)S_{V}=\frac{ap_{H}I_{H}}{N_{H}}S_{V},$$

the NBD transmission functions approximate that used in the corresponding homogeneous mixing models [37] (Equations (1) and (2)) when the parameter *k* approximates +∞. Therefore, the homogeneous mixing models can be viewed as special cases of the NBD model (Equation (5)).

Based on the next-generation matrix operator approach [43], the basic reproductive number, $R_{0}$, was derived (see details in Appendix A):

$$R_{0}=\sqrt{\frac{N_{V}}{N_{H}}\frac{a^{2}p_{H}p_{V}\delta_{H}\delta_{V}}{\mu_{V}\left(\delta_{H}+\mu_{H}\right)\left(\gamma_{H}+\mu_{H}\right)\left(\delta_{V}+\mu_{V}\right)}}.$$

It is identical to the $R_{0}$ in the corresponding homogeneous mixing models [38]. Note that it is independent of *k*, indicating that $R_{0}$ is not dependent on the level of heterogeneity. This result agrees with previous works [24,44], in which the authors inferred the maximum likelihood estimation of $R_{0}$ and the dispersion parameter *k* that characterized heterogeneity, and it was shown that the maximum likelihood estimation of $R_{0}$ was independent of *k*, although the associated confidence intervals do depend on it. The independence can be explained by $R_{0}$ being an average value fundamentally, neglecting heterogeneity. In this way, the disease outbreaks cannot be fully understood if individual variation in infectiousness is neglected [23], considering the transmission variance. Therefore, heterogeneity is indispensable for modeling transmission dynamics.

### 2.3. Extended Model

In reality, the number of mosquitoes is not constant but varies with the climate [45,46,47,48], environment [49], and human behavior [50]. Climate influences the transmission of DF by affecting mosquito development and mosquito/human interactions [45]. The entire lifecycle of the mosquito, including breeding, development and mortality, is influenced by temperature [45,46,47,48]. Temperature also influences virus replication and transmission. Studies have found that high temperature can increase the rate of viral replication within the vector and therefore shorten the EIP [41,51]. Precipitation also influences the mosquito population by providing habitats for their aquatic stages [45,47]. It has been shown that higher precipitation is associated with increased mosquito populations [47,52]. However, intense rainfall may wash out breeding sites and therefore have a negative effect on mosquito populations [45,52]. The mosquito population is also influenced by human behavior since man-made containers can provide habitats for the aquatic stages of mosquitoes [52,53,54,55].

**Figure 5:**
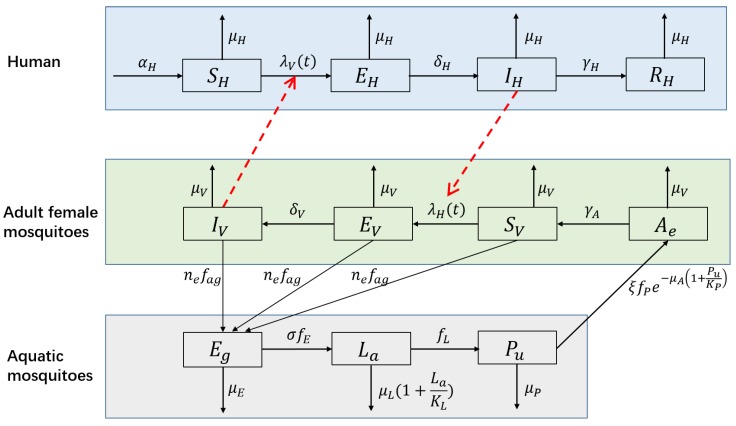
Flow diagram of the extended model with the aquatic phase of mosquitoes.

When modeling dengue dynamics, it was necessary to consider the above factors. First, we extended the NBD SEIR-SEI model (Equation (5)) by explicitly modeling the aquatic immature life stages of mosquitoes. Four additional compartments, the eggs ($E_{g}$), larva ($L_{a}$), pupae ($P_{u}$) and the emerging females that are unable to reproduce ($A_{e}$), were added to the above model. Figure 5 presents the structure of the extended model, and the definition for each symbol in this figure is shown in Table 2. Each life-stage was represented by an ordinary differential equation (the fifth to the eighth equation in Equation (6)). The adults that are able to mate and feed ($S_{V}$, $E_{V}$ and $I_{V}$) can oviposit and begin their life cycle [56]. Density-dependent mortality was assumed at the larval stage [47,57] because inter- and intra-specific competition (e.g., for food) at the larvae stage usually limits mosquito populations [58]. Because mosquito pupae do not feed and rely solely on energy stored from the larval stage [59], the proportion of emerging pupae that survives to emergence transits to stage ’emerging adults’. Pupa density-dependent success of adult emergence was assumed [47,57] as emergence success has been shown to be negatively correlated to pupa density [52,60]. Second, the constant parameters used in Equation (5), which were influenced by climate and the environment, were replaced by climate- and/or environment-dependent functions. These parameters included the oviposition rate, mortality and development rate of mosquitoes; the biting frequency; and EIP. The impacts of the environment and human activities on the mosquito population were characterized by the carrying capacity for immature mosquitoes ($\pi_{max}$). It was related to the water level, which increased due to rainfall. However, if there was an extreme rainfall event and the water level was close to the maximum water level, then a spillover effect was triggered, in which a fraction of the immature mosquitoes were washed out from their breeding sites [19,31]. The form and coefficients of the climate- and/or environment-dependent functions were based on [19,31,47]. The detailed forms of these functions can be found in Appendix B. The extended model was represented by the set of differential equations shown in Equation (6):

(6) $$\left\{\begin{array}{cc} \frac{dS_{H}}{dt} & =\alpha_{H}N_{H}-k\ln\left(1+\frac{bp_{V}I_{V}}{kN_{H}}\right)S_{H}-\mu_{H}S_{H},\\ \frac{dE_{H}}{dt} & =k\ln\left(1+\frac{ap_{V}I_{V}}{kN_{H}}\right)S_{H}-\left(\delta_{H}+\mu_{H}\right)E_{H},\\ \frac{dI_{H}}{dt} & =\delta_{H}E_{H}-\left(\gamma_{H}+\mu_{H}\right)I_{H},\\ \frac{dR_{H}}{dt} & =\gamma_{H}I_{H}-\mu_{H}R_{H},\\ \frac{dE_{g}}{dt} & =n_{E}f_{ag}N_{V}-\sigma f_{E}E_{g}-\mu_{E}E_{g},\\ \frac{dL_{a}}{dt} & =\sigma f_{E}E_{g}-f_{L}L_{a}-\mu_{L}\left(1+\frac{L_{a}}{K_{L}}\right)L_{a},\\ \frac{dP_{u}}{dt} & =f_{L}L_{a}-f_{P}P_{u}-\mu_{P}P_{u},\\ \frac{dA_{e}}{dt} & =\xi f_{P}P_{u}\exp\left(-\mu_{A}\left(1+\frac{P_{u}}{K_{P}}\right)\right)-\gamma_{A}A_{e}-\mu_{V}A_{e},\\ \frac{dS_{V}}{dt} & =\gamma_{A}A_{e}-k\ln\left(1+\frac{bp_{H}I_{H}}{kN_{H}}\right)S_{V}-\mu_{V}S_{V},\\ \frac{dE_{V}}{dt} & =k\ln\left(1+\frac{ap_{H}I_{H}}{kN_{H}}\right)S_{V}-\left(\delta_{V}+\mu_{V}\right)E_{V},\\ \frac{dI_{V}}{dt} & =\delta_{V}E_{V}-\mu_{V}I_{V}. \end{array}\right.$$

### 2.4. Model Calculation

To reproduce the 2014 DF outbreak in Guangzhou, we calibrated the extended model (Equation (6)) to the daily new DF cases data of that outbreak. A number of parameters were estimated using the GlobalSearch algorithm in the MATLAB Global Optimization Toolbox [62,63]. The normalized root mean square error (NRMSE) [64] was chosen as the goodness of fit between the model output and the referenced daily new DF cases, as well as the objective function of the model fitting procedure. NRMSE varied from −∞ (poor fit) to 1 (perfect fit) [64].

The extended model developed here is a type of deterministic mathematical dynamics model. Therefore, the introduction of an infectious individual to the original disease-free population would result in an epidemic outbreak [65]. During the initial phase of the 2014 DF outbreak in Guangzhou, only sporadic cases occurred. Each of these cases had the chance to infect mosquitoes and then trigger the outbreak, while he or she also had the possibility of infecting no mosquitoes, thereby breaking the chain of transmission. To our best knowledge, no study showed which case triggered the overall outbreak. To better reproduce the overall outbreak, i.e., to better fit the daily new DF cases shown in Figure 2, we ignored this initial phase and only fit the data from 15 July 2014, to the end of the outbreak. We chose this period because from that day on, the daily new DF cases showed a clear increasing trend (Figure 2). To describe the initial trigger, we employed an unknown parameter, $t_{0}$, to denote the date on which a trigger individual was infected. This was realized by setting $I_{H}\left(t\right)=0$ when $t<t_{0}$ and $I_{H}\left(t_{0}\right)=1$.

The 2014 DF outbreak in Guangzhou peaked on October 1, with 1613 new cases. After that day, the daily new DF cases showed a notable decrease (Figure 2). This decrease in cases may have been caused by the interventions, including larval breeding eradication, killing of adult mosquitoes with pesticides, public health education and community involvement [66]. Here, we separated the entire transmission process into two phases. In the first phase, an exponential growth phase was observed, during which interventions played no or a negligible role. When the interventions began to take effect, the transmission process entered the second phase. The date separating the two phases was set to 23 September 2014, using the method presented in [67]. The mortality of the mosquitoes must be different in the two phases. However, it was difficult to estimate the mortality caused by interventions. Here, rather than estimating the mortality, we modeled the two phases with different heterogeneity levels, reflected by different parameters, $k^{1}$ and $k^{2}$ ($k^{2}<k^{1}$). The smaller $k^{2}$ reflected a higher degree of heterogeneity in the second phase. We can image that the mosquitoes eradicated by human interventions were still “alive”, but they lost the ability to bite humans. Therefore, fewer people had the chance of being bitten, and the level of heterogeneity increased. In the model, this was realized by setting $k=k^{1}$ before September 23, and after that day, setting $k=k^{2}$.

To solve Equation (6), initial values for all the compartments were needed. Since the mosquito population for only the first simulated year was affected by the initial value for eggs [31,47,68], we ran the model over the period 2013 to 2014, although our focus was to simulate the dengue outbreak in 2014. The year 2013 was used to achieve a mosquito population for 2014 that was not sensitive to the initial conditions. Here, we set $E_{g}\left(0\right)=1000$ and $L_{a}\left(0\right)=1$, and the initial values of all the other mosquito compartments were set to be zero. Vertical transmission was not considered here; thus, we ignored the DF cases in 2013 for simplicity. We set $S_{H}\left(0\right)=12.8389\times 10^{6}$, which was the size of the permanent population at the end of the year 2012, and $E_{H}\left(0\right)=I_{H}\left(0\right)=R_{H}\left(0\right)=0$.

During the transmission process of DF, the mild or asymptomatic infections cannot be ignored [69]. It was estimated that the overall inapparent-to-symptomatic (I:S) ratio was 2.2:1 (95% CI: 1.1-4.2:1) for DF in Zhongshan City [69], which is adjacent to Guangzhou. For simplicity, we used the daily DF cases multiplied by 3.2 to fit the model output.

In addition to the three unknown parameters introduced above ($t_{0}$, $k^{1}$ and $k^{2}$), five more unknown parameters were estimated: (1) the transmission probability from vector to human per bite ($p_{V}$), (2) the transmission probability from human to vector per bite ($p_{H}$), (3) the reciprocal of the intrinsic incubation period ($\delta_{H}$), (4) the reciprocal of the infectious period for humans ($\gamma_{H}$), and (5) the carrying capacity for immature mosquitoes ($\pi_{max}$). The ranges for parameters (1)–(4) are shown in Table 1. Eight parameters in total were estimated during the calibration process. Some other parameters that were used are shown in Appendix B.

## 3. Results

### 3.1. Transmission Dynamics with Different Heterogeneity Levels

**Figure 6:**
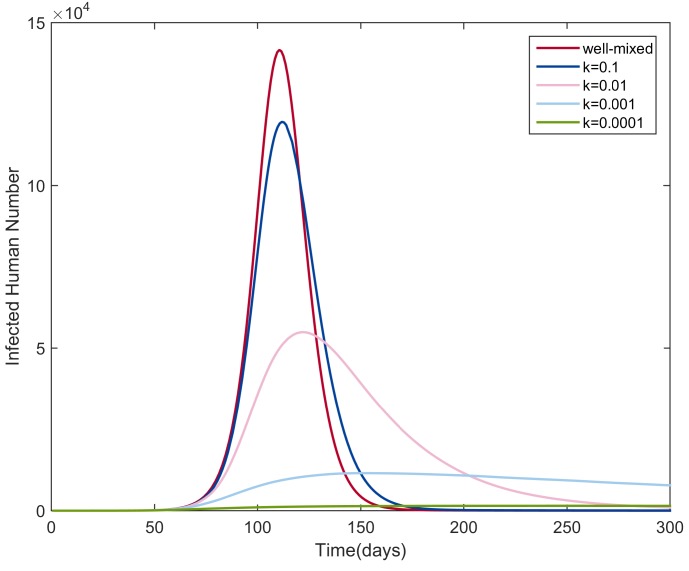
Infection curves for different values of *k* for the NBD SEIR-SEI model and the corresponding well-mixed model.

Through numerical simulations, we explored the influence of the heterogeneity of mosquito bites of humans on the transmission dynamics. We simulated the NBD SEIR-SEI model (Equation (5)) with different values for *k*: 0.1, 0.01, 0.001, and 0.0001. As the value of *k* decreases, the heterogeneity level increases. For all simulations, we assumed an equal amount of humans and mosquitoes, i.e., $N_{H}=N_{V}$. We set the initial conditions as follows: $N_{H}=10^{6}$, $I_{H}\left(0\right)=1$, $E_{H}\left(0\right)=R_{H}\left(0\right)=0$, $S_{H}\left(0\right)=N_{H}-E_{H}\left(0\right)-I_{H}\left(0\right)-R_{H}\left(0\right)$, $S_{V}\left(0\right)=N_{V}$, and $E_{V}\left(0\right)=I_{V}\left(0\right)=0$. The parameter values were set to the values in Table 1. The simulation results are shown in Figure 6. For comparison, the epidemic curve predicted by the corresponding homogeneous mixing model was also drawn.

As the level of heterogeneity increased (the value of *k* decreased), the gap between the infection curves from the NBD SEIR-SEI model and that from the homogeneous mixing model increased and the peak size decreased. We explained these results by imagining a scenario with a high level of heterogeneity, in which only a small proportion of people had the chance of being bitten by infectious mosquitoes and being infected while most of people avoided being bitten and infected. This resulted in a smaller transmission rate and a smaller peak size compared with the output from the homogeneous mixing model. In real DF outbreaks, human hosts and mosquitoes are heterogeneously mixed. A higher degree of heterogeneity resulted in a higher extinction probability of diseases [23].

### 3.2. Model Calibration Results

**Figure 7:**
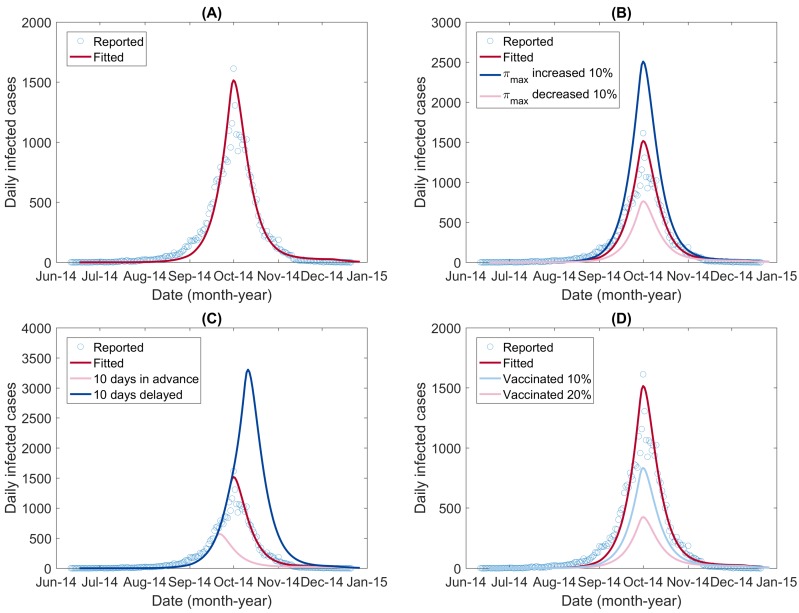
The epidemic curves fitted and simulated for DF in different scenarios. (**A**) the daily new DF cases in Guangzhou, 2014, and the fitted epidemic curve by the extended model; (**B**) simulated epidemic curves assuming that the carrying capacity for immature mosquitoes ($\pi_{max}$) was increased or decreased by 10%; (**C**) simulated epidemic curves assuming that the interventions were taken ahead or delayed by 10 days; and (**D**) simulated epidemic curves assuming that 10% or 20% of the population had been vaccinated and were immune to DENV.

Figure 7 (A) shows the reported daily new DF cases and the fitted epidemic curve. The value of NRMSE, which determined the goodness of fit, was 0.7299. The optimized values of the unknown parameters are listed in Table 3. Because of the intrinsic stochasticity of the GlobalSearch algorithm [62,63], slightly different results may be obtained when running the programs. With random values within the range of the parameters (Table 1 and assumed for the others), we ran the optimal program multiple times and obtained the standard derivation of each parameter.

Both $k^{1}$ and $k^{2}$ were small, indicating that there was high heterogeneity during the entire outbreak period. The large difference between $k^{1}$ and $k^{2}$ ($k^{2}<k^{1}$) indicated that the heterogeneity level in the second phase was considerably higher than that in the first stage. We believe that this result may be the combined effect of human interventions, climate conditions and enhancement of human protection.

### 3.3. Simulation of Dengue Transmission in Different Scenarios

We simulated the impact of interventions on the spread of DF in different scenarios. The epidemic curves were plotted by changing one parameter while keeping the others identical to the values fitted to the 2014 DF outbreak in Guangzhou.

First, we assumed that the carrying capacity for immature mosquitoes ($\pi_{max}$) was increased or decreased by 10% and then simulated the DF spread in these two scenarios. The simulated epidemic curves are shown in Figure 7B. The simulation results showed that the outbreak would reach the peak at the same time but with different peak sizes: an increased carrying capacity resulted in a higher peak size, whereas a decreased carrying capacity resulted in a lower peak size. The total number of infections was also influenced, increased by 63.47% or decreased by 50.29%, corresponding to $\pi_{max}$ increasing or decreasing, respectively (shown in Table 4).

Second, we assumed that the beginnings of the interventions were set earlier or delayed by 10 days. Here, we assumed that the much smaller $k^{2}$ compared with $k^{1}$ mainly resulted from human interventions. We estimated that the date that separated the two phases was 23 September for the 2014 DF outbreak in Guangzhou. To simulate the impact of the timing of interventions, we set the timing of the switch from $k^{1}$ to $k^{2}$ to be September 13 and October 3, respectively. The simulated epidemic curves are shown in Figure 7C. Both the peak time and the total number of infections were changed. The transmission was observed to peak early with a lower peak size when the timing of the switch from $k^{1}$ to $k^{2}$ was set earlier by 10 days. In contrast, if the timing was delayed by 10 days, the daily infected cases would continue to increase until it reached a much higher peak (Figure 7C). The total number of infections in the two scenarios decreased by 60.69% and increased by 124.59%, respectively (Table 4).

Third, we assessed the impact of dengue immunization programs if implemented in Guangzhou in the future. The dengue vaccine Dengvaxia (also referred to as CYD-TDV) has been licensed and registered in several countries [70]. Here, we assumed that a dengue vaccination program had been implemented in Guangzhou and that, among the population, proportions of 10% and 20% had been vaccinated and were immune to DENV. We simulated the transmission in these two scenarios by decreasing the initial value of the susceptible humans, $S_{H}\left(0\right)$, by 10% and 20%, respectively, and increased the initial value of the immune humans, $R_{H}\left(0\right)$, by the corresponding proportion. The simulated epidemic curves are shown in Figure 7D. The simulation results showed that, as the proportion of the effectively vaccinated population increased, the peak size became smaller and the total number of infections decreased (Table 4). In practice, it is necessary to consider the cost efficacy of the vaccination program.

## 4. Discussion

In this paper, a mathematical model with nonlinear NBD transmission functions was developed to analyze the impact of heterogeneity of mosquito bites of humans during the dengue transmission process. The simulation results suggested that a higher level of heterogeneity can result in a small peak size and a smaller number of total infections. The simulations in different scenarios suggested introducing interventions early and clearing the mosquito breeding environment to prevent and control the transmission of DF.

It has been emphasized in many previous studies that the heterogeneity should be taken into account to model the transmission of DF [9,71,72]. Studies have indicated that people, rather than mosquitoes, influence the spatial dynamics of dengue virus [71,73]. Favier et al. modeled the contact heterogeneity using a stochastic SEIR-SEI model and the probability for hosts visiting others in another house [71]. Cosner et al. used a discrete-time multi-patch model to model the effects of the mobility of people on DF transmission dynamics [15,16]. Agent-based, spatially explicit models were applied to understand the factors contributing to DF spread, to explore the impacts of mosquito movement and distribution on DF transmission, to find optimized dengue control strategies, and to estimate the effectiveness and cost effectiveness of future vaccination programs [11,18,19,74].

The NBD SEIR-SEI model developed in this study was novel in terms of the use of the NBD transmission functions, which characterized the heterogeneity of mosquito bites of humans by assuming that the encounters between humans and mosquitoes were dispersed. It is known that most female *Ae. aegypti* may spend their lifetime in or around the houses where they emerge as adults [73,75]. Thus, for a disease-free population, a person infected with DENV can only spread DENV to the mosquitoes near the position he or she had appeared. Similarly, only a small portion of humans had the chance of being infected by one specific infectious mosquito. Therefore, the rate of DF transmission was not as fast as that predicted by the homogeneous mixing models, nor was the transmission intensity. Whereas the homogeneous mixing model always predicted that almost all people were infected by the dengue virus after an epidemic [37], the NBD SEIR-SEI model can reproduce a real dengue epidemic well. The very small fitted $k^{1}$ and $k^{2}$ values indicated that it was indeed highly heterogeneous. Therefore, it was necessary to consider the influence of heterogeneity when modeling the dynamics of DF.

The extended model including the aquatic phase of immature mosquitoes and integrating the influence of the environment and climate factors on them was used to reproduce the 2014 DF outbreak in Guangzhou. The fitted results (Figure 7A) demonstrated the ability of the model to describe the real DF transmission processes. It was also used to simulate dengue transmission dynamics in different scenarios and to evaluate the effects of different interventions quantitatively. The simulation results showed that performing interventions as early as possible and decreasing the mosquito carrying capacity were necessary to prevent and control DF outbreaks. Therefore, two important issues should be addressed. The first is to develop an early warning system to predict the risk of a dengue epidemic. Once the risk is higher than a predefined ‘alert’ threshold, intervention strategies should be started and performed in a timely manner. The other issue is to decrease the carrying capacity of aquatic phase of mosquitoes, for example, by cleaning water containers and other egg-laying habitats.

As we know, the more sophisticated the model is, the more data that need to be collected and the higher the requirements on the parametrization. Compared to the meta-population and agent-based models [11,15,16,18,19,74], the NBD SEIR-SEI model was considerably more tractable in that it used only one parameter to characterize the heterogeneity of host-vector encounters. However, the modeling assumptions deserve further exploration. In future research, it would be interesting to integrate the NBD model with other methods for a greater understanding of the DF’s spatiotemporal diffusion.

## 5. Conclusions

DF in Guangdong Province in China has been a major public health concern. Understanding the spread of DF is key to risk assessment and performing preventive interventions. In this study, we proposed a dynamic mathematical model with NBD transmission functions to analyze the influence of the heterogeneity of mosquito bites of humans during the DF transmission process. The results showed a smaller peak size and a smaller number of total infections with a higher level of heterogeneity. These findings contribute to a better understanding of DF transmission dynamics. The presented model can also be modified to describe the transmission dynamics of other vector-borne diseases.

## Figures and Tables

**Table 1:** Parameter notation, biological meaning, values and sources.

Parameter	Biological Meaning	Range	Value	Source
*a*	Average daily biting rate	0.3–1	1	[8]
$p_{V}$	Transmission probability from vector to human per bite	0.1–0.75	0.5	[8]
$p_{H}$	Transmission probability from human to vector per bite	0.5–1	0.75	[8]
$1/\mu_{H}$	Human life expectancy (years)	-	75	Assumed
$\mu_{V}^{-1}$	Average lifespan of mosquitoes (days)	4–50	21	[8]
$\delta_{H}^{-1}$	Intrinsic incubation period (IIP, days)	4–10	7	[41]
$\delta_{V}^{-1}$	Extrinsic incubation period (EIP, days)	8–12	10	[8]
$\gamma_{H}^{-1}$	Infectious period (days)	1–7	4.5	[42]

**Table 2:** Parameter notation, biological meaning and values used in the extended model.

Parameter	Biological Meaning	Values ${}^{1}$
σ	Diapause	1 in Mar. 15 to Oct. 25; 0 otherwise ([31])
$n_{E}$	Eggs per gonotrophic cycle (per female)	Temperature dependent
$f_{ag}$	1/duration for gonotrophic cycle (per day)	Temperature dependent
$f_{E}$	Egg development rate	Temperature and precipitation dependent
$f_{L}$	Larva development rate	Temperature and precipitation dependent
$f_{P}$	Development rate of pupae to emerging adults	Temperature dependent
$\mu_{E}$	Egg mortality rate	0.05 ([47])
$\mu_{L}$	Mortality for larva	Temperature and density dependent
$\mu_{P}$	Mortality for pupa	Temperature dependent
$\mu_{A}$	Mortality rate during adult emergence	0.1 ([47])
$\mu_{V}$	Mortality rate of adult mosquitoes	Temperature dependent
ξ	Sex ratio of *Aedes albopictus* at emergence	0.5 ([61])
$\gamma_{A}$	Development rate of emerging adults (day${}^{-1}$)	0.4 ([47])
$K_{L}$	Carrying capacity of mosquito larvae population	Precipitation and environment dependent
$K_{P}$	Carrying capacity of mosquito pupae population	Precipitation and environment dependent
$\pi_{max}$	Maximum carrying capacity for immature mosquitoes	Environment and density dependent
$N_{V}$	Total population of adult female mosquitoes	$N_{V}=S_{V}+E_{V}+I_{V}$

${}^{1}$ The details for the climate- and/or environment-dependent functions are shown in Appendix B.

**Table 3:** Parameter notation, biological meaning, and optimal values.

Parameter	Biological Meaning	Optimized Value ± Std ${}^{1}$
$t_{0}$	The date on which an infectious human trigged the outbreak	17 June 2014 ± 7 days
$k^{1}$	Heterogeneity level in the 1st phase	$3.8263\times 10^{-4}\pm 1.1316\times 10^{-6}$
$k^{2}$	Heterogeneity level in the 2nd phase	$2.1379\times 10^{-7}\pm 6.6802\times 10^{-9}$
$p_{V}$	Transmission probability from vector to human per bite	0.414 ±0.012
$p_{H}$	Transmission probability from human to vector per bite	0.682 ±0.019
$\pi_{max}$	Maximum carrying capacity of immature mosquitoes	$5.1354\times 10^{6}$± 14665
$\delta_{H}^{-1}$	Intrinsic incubation period (IIP, days)	7.853 ± 0.22
$\gamma_{H}^{-1}$	Infectious period (days)	4.746 ± 0.135
	Estimated cumulative reported cases	37,733

${}^{1}$ std refers to the standard deviation of each parameter through 100 runs.

**Table 4:** The total number of infections reported and simulated in assumed scenarios.

Scenario	Reported Total Infections ${}^{1}$	Change ${}^{2}$
Reported	37,420	-
Fitted	37,733	-
$\pi_{max}$ decreased by 10%	18,758	Decreased by 50.29%
$\pi_{max}$ increased by 10%	61,684	Increased by 63.47%
Intervention 10 days earlier	14,831	Decreased by 60.69%
Intervention 10 days delayed	84,746	Increased by 124.59%
Vaccinating 10%	21,232	Decreased by 43.73%
Vaccinating 20%	11,563	Decreased by 69.36%

${}^{1}$ The reported total infections were calculated by multiplying the total infections predicted by the models by a constant 3.2 according to the I:S ratio [69]; ${}^{2}$ The rates of change were calculated based on the fitted total infection.

## Abbreviations

The following abbreviations were used in this manuscript:

DF	dengue fever
DENV	dengue virus
NBD	negative binomial distribution
I:S	inapparent-to-symptomatic ratio

## Appendix A. The Basic Reproductive Number

Based on the next-generation matrix operator approach [43], we derive the basic reproductive number, $R_{0}$. First, the compartments are sorted, $x=(E_{H},E_{V},I_{H},I_{V},S_{H},S_{V},R_{H})$, such that the infected individuals are contained in the first *m* compartments. Here, m=4, and $E_{H}$, $E_{V}$, $I_{H}$, $I_{V}$ are the first *m* compartments. The disease-free equilibrium can be obtained, $x_{0}=\left(0,0,0,0,N_{H},N_{V},0\right)$.

Then, the derivatives of the compartments are decomposed as $\dot{x_{i}}=F_{i}-V_{i}$, where

$$F=\left(\begin{array}{c} k\ln\left(1+\frac{ap_{V}I_{V}}{kN_{H}}\right)S_{H}\\ k\ln\left(1+\frac{ap_{H}I_{H}}{kN_{H}}\right)S_{V}\\ 0\\ 0\\ \vdots \end{array}\right),\;V=\left(\begin{array}{c} \left(\delta_{H}+\mu_{H}\right)E_{H}\\ \left(\delta_{V}+\mu_{V}\right)E_{V}\\ -\delta_{H}E_{H}+\left(\gamma_{H}+\mu_{H}\right)I_{H}\\ -\delta_{V}E_{V}+\mu_{V}I_{V}\\ \vdots \end{array}\right).$$

Next, the next-generation matrix (NGM) can be obtained,

$$FV^{-1}=\left(\begin{array}{cccc} 0 & \frac{ap_{V}\delta_{V}}{\mu_{V}\left(\delta_{V}+\mu_{V}\right)} & 0 & \frac{ap_{V}}{\mu_{V}}\\ \frac{ap_{H}\delta_{H}N_{V}}{\left(\delta_{H}+\mu_{H}\right)\left(\gamma_{H}+\mu_{H}\right)N_{H}} & 0 & \frac{ap_{H}N_{V}}{\left(\gamma_{H}+\mu_{H}\right)N_{H}} & 0\\ 0 & 0 & 0 & 0\\ 0 & 0 & 0 & 0 \end{array}\right),$$

where

$$F=\left[\frac{\partial F_{i}}{\partial x_{j}}\left(x_{0}\right)\right]={\left(\begin{array}{cccc} 0 & 0 & 0 & \frac{kap_{V}S_{H}}{kN_{H}+ap_{V}I_{V}}\\ 0 & 0 & \frac{kap_{H}S_{V}}{kN_{H}+ap_{H}I_{H}} & 0\\ 0 & 0 & 0 & 0\\ 0 & 0 & 0 & 0 \end{array}\right)}_{x=x_{0}}=\left(\begin{array}{cccc} 0 & 0 & 0 & ap_{V}\\ 0 & 0 & \frac{ap_{H}N_{V}}{N_{H}} & 0\\ 0 & 0 & 0 & 0\\ 0 & 0 & 0 & 0 \end{array}\right),$$

$$V=\left[\frac{\partial V_{i}}{\partial x_{j}}\left(x_{0}\right)\right]=\left(\begin{array}{cccc} \delta_{H}+\mu_{H} & 0 & 0 & 0\\ 0 & \delta_{V}+\mu_{V} & 0 & 0\\ -\delta_{H} & 0 & \gamma_{H}+\mu_{H} & 0\\ 0 & -\delta_{V} & 0 & \mu_{V} \end{array}\right),\;1\leq i,j\leq m.$$

Thus, the spectral radius of the NGM is the basic reproductive number $R_{0}$

$$R_{0}=\sqrt{\frac{N_{V}}{N_{H}}\frac{a^{2}p_{H}p_{V}\delta_{H}\delta_{V}}{\mu_{V}\left(\delta_{H}+\mu_{H}\right)\left(\gamma_{H}+\mu_{H}\right)\left(\delta_{V}+\mu_{V}\right)}}.$$

## Appendix B. The Parameters Dependent on Temperature and Precipitation

Temperature can influence the oviposition, mortality and transition rates for different stages of the life-cycle of the mosquito. Precipitation can provide habitats for the aquatic phase of mosquitoes; however, intense rainfall may wash out breeding sites and cause a reduction in the mosquito population [45]. The mosquito population was also influenced by the environment. In this section, we list the forms of climate- and/or environment-dependent functions. These forms and coefficients were referenced from [19,31,47].

### Appendix B.1. Eggs per Gonotrophic Cycle

The number of eggs per gonotrophic cycle ($n_{e}$, day${}^{-1}$) was temperature dependent:

$$n_{e}=\max\left(-0.5717T_{t}^{2}+31.8313T_{t}-349.8819,0\right),$$

where $T_{t}$ denotes the mean temperature (${}^{{}^{\circ}}$K) on day *t*.

### Appendix B.2. Reciprocal of the Duration for Gonotrophic Cycle

The reciprocal of the duration for gonotrophic cycle ($f_{ag}$, day${}^{-1}$) was temperature dependent:

$$f_{ag}=24\times \frac{0.0102\times \left(T_{t}/298\right)\times e^{\frac{60513.2}{R}\left(\frac{1}{298}-\frac{1}{T_{t}}\right)}}{1+e^{\frac{705550}{R}\left(\frac{1}{308.352}-\frac{1}{T_{t}}\right)}},$$

where *R* is the universal gas constant (1.987 cal mol${}^{-1}$deg${}^{-1}$).

### Appendix B.3. Water Level

The environmental carrying capacity for the aquatic phase of mosquitoes was associated with the water level, which was assumed to be fluctuating between a minimum ($\omega_{min}$) and a maximum ($\omega_{max}$) level. The daily water level was determined by the precipitation, evaporation, and the maximum and minimum water levels:

$$\omega \left(t+1\right)=\left\{\begin{array}{cc} \omega (t)-EV(t), & \omega \left(t\right)+RF\left(t\right)-EV\left(t\right)>\omega_{max},\\ \omega (t)+RF(t)-EV(t), & \omega_{min}<\omega \left(t\right)+RF\left(t\right)-EV\left(t\right)<\omega_{max},\\ \omega (t)+RF(t), & \omega \left(t\right)+RF\left(t\right)-EV\left(t\right)<\omega_{min}, \end{array}\right.$$

where

ω: daily water level (mm);

$\omega_{max}$: maximum water level, beyond which the potential breeding sites for the mosquito will overflow (mm), and here, $\omega_{max}=778$ [31];

$\omega_{min}$: minimum water level, which represents water containers and other standing water shielded from evaporation (mm), and here, $\omega_{min}=400$ [31];

EV(t): daily evaporation on day *t* (mm);

RF(t): daily rainfall on day *t* (mm).

### Appendix B.4. Spillover Effect

Heavy rains, which were defined as daily cumulative precipitation greater than 50 mm [31], can wash out a fraction of η of the immature mosquitoes from their breeding sites, such as water containers:

$$\eta =\eta_{0}\times \frac{1.2\times {\left(\frac{\omega }{\omega_{max}}\right)}^{20}}{1+1.2\times {\left(\frac{\omega }{\omega_{max}}\right)}^{20}},$$

where

$\eta_{0}$: the maximum heavy rain washout fraction, and here, $\eta_{0}=0.465$ [31].

### Appendix B.5. Egg Development Rate

The egg development rate ($f_{E}$, day${}^{-1}$) was temperature and precipitation dependent:

$$f_{E}=\left(1-\theta \right)f_{E,ideal}\times \frac{20{\left(\frac{\omega }{\omega_{max}}\right)}^{8}}{1+20{\left(\frac{\omega }{\omega_{max}}\right)}^{8}}+f_{E,ideal}*\theta ,$$

where

θ: the ratio of minimum egg hatching rate to ideal egg hatching rate (day${}^{-1}$), and here, θ=0.684 [31];

$f_{E,ideal}$: the ideal development rate for eggs

$$f_{E,ideal}=24\times \frac{0.00835\times \left(T_{t}/298\right)\times e^{\frac{46701.2}{R}\left(\frac{1}{298}-\frac{1}{T_{t}}\right)}}{1+e^{\frac{309796.0}{R}\left(\frac{1}{313.511}-\frac{1}{T_{t}}\right)}}.$$

### Appendix B.6. Larva Development Rate

The larva development rate ($f_{L}$, day${}^{-1}$) was temperature and density dependent:

$$f_{L}=\left(1-\lambda \right)f_{L,ideal}\times \frac{2{\left(\frac{L_{a}}{K_{L}}\right)}^{-1}}{1+2{\left(\frac{L_{a}}{K_{L}}\right)}^{-1}}+\lambda *f_{L,ideal},$$

where

$f_{L,ideal}$: the ideal development rate for larva (day${}^{-1}$):

$$f_{L,ideal}=24\times \frac{0.00608\times \left(T_{t}/298\right)\times e^{\frac{51681.3}{R}\left(\frac{1}{298}-\frac{1}{T_{t}}\right)}}{1+e^{\frac{186888.0}{R}\left(\frac{1}{313.208}-\frac{1}{T_{t}}\right)}},$$

$L_{a}$: number of larva;

$K_{L}$: carrying capacity of mosquito larvae population, $K_{L}=\pi_{max}\times \frac{\omega }{\omega_{max}}$;

$\pi_{max}$: maximum carrying capacity for immature stages, to be estimated;

λ: the ratio of minimum larvae development rate to ideal larvae development rate (day${}^{-1}$), and here, λ=0.598 [31].

### Appendix B.7. Mortality Rate for Larva

The mortality rate for larva ($\mu_{L}$, day${}^{-1}$) was temperature and density dependent:

$$\mu_{L}=\mu_{L,ideal}\left(1+\frac{L_{a}}{K_{L}}\right),$$

where $\mu_{L,ideal}$ is the ideal mortality rate for larva:

$$\mu_{L,ideal}=\left\{\begin{array}{cc} 0.0000866T_{t}^{2}-0.00368T_{t}+0.0451, & T_{t}\geq 12.5{\;}^{{}^{\circ}}C,\\ 0.5, & T_{t}<12.5{\;}^{{}^{\circ}}C. \end{array}\right.$$

### Appendix B.8. Development Rate of Pupae to Emerging Adults

The development rate of pupae to emerging adults ($f_{P}$, day${}^{-1}$) was temperature dependent:

$$f_{P}=24\times \frac{0.0143\times \left(T_{t}/298\right)\times e^{\frac{44093.2}{R}\left(\frac{1}{298}-\frac{1}{T_{t}}\right)}}{1+e^{\frac{100261}{R}\left(\frac{1}{330.058}-\frac{1}{T_{t}}\right)}}.$$

### Appendix B.9. Mortality Rate for Pupae

The mortality rate for pupa ($\mu_{P}$, day${}^{-1}$) was temperature dependent:

$$\mu_{P}=\left\{\begin{array}{cc} 0.01, & 12.5{\;}^{{}^{\circ}}C\leq T_{t}\leq 35{\;}^{{}^{\circ}}C,\\ 0.5, & else. \end{array}\right.$$

### Appendix B.10. Death Rate for Adults

The death rate for adults ($\mu_{V}$, day${}^{-1}$) was temperature dependent:

$$\mu_{V}=\left\{\begin{array}{cc} 0.000114T_{t}^{2}-0.00427T_{t}+0.0639, & T_{t}\geq 15{\;}^{{}^{\circ}}C,\\ 0.5, & else. \end{array}\right.$$

### Appendix B.11. Extrinsic Incubation Period

The EIP ($\frac{1}{\delta_{V}}$, days) was temperature dependent:

$$\frac{1}{\delta_{V}}=24\times \frac{0.00333\times \left(T_{t}/298\right)\times e^{\frac{70802.6}{R}\left(\frac{1}{298}-\frac{1}{T_{t}}\right)}}{1+e^{\frac{177239}{R}\left(\frac{1}{448.619}-\frac{1}{T_{t}}\right)}}.$$

### Appendix B.12. Biting Rate

The biting rate (*b*, day${}^{-1}$) was temperature dependent:

$$b=\max(-0.004981T^{2}+0.274T-2.94,0).$$
